# Rural-urban difference in the prevalence of hypertension in West Africa: a systematic review and meta-analysis

**DOI:** 10.1038/s41371-022-00688-8

**Published:** 2022-04-16

**Authors:** Ruqayya Nasir Sani, Paul J. Connelly, Mette Toft, Neneh Rowa-Dewar, Christian Delles, Danijela Gasevic, Kamilu Musa Karaye

**Affiliations:** 1 Department of Medicine, Aminu kano Teaching Hospital, Kano, Nigeria; 2 Center for Global Health, Usher Institute, The University of Edinburgh, Edinburgh, UK; 3 Institute of Cardiovascular & Medical Sciences, University of Glasgow, Glasgow, UK; 4 School of Public Health and Preventive Medicine, Monash University, Melbourne, Victoria Australia; 5 Bayero University Kano, Kano, Nigeria

**Keywords:** Hypertension, Disease prevention, Risk factors

## Abstract

Urbanisation is considered a major contributor to the rising prevalence of hypertension in West Africa, yet the evidence regarding rural-urban differences in the prevalence of hypertension in the region has been mixed. A systematic literature search of four electronic databases: PubMed, Embase, African Journals Online, and WHO’s African Index Medicus; and reference lists of eligible studies was carried out. Original quantitative studies describing the rural-urban difference in the prevalence of hypertension in one or more countries in West Africa, and published in English language from the year 2000 to 2021 were included. A random effects meta-analysis model was used to estimate the odds ratio of hypertension in rural compared to urban locations. A limited sex-based random effects meta-analysis was conducted with 16 studies that provided sex-disaggregated data. Of the 377 studies screened, 22 met the inclusion criteria (*n* = 62,907). The prevalence of hypertension was high in both rural, and urban areas, ranging from 9.7% to 60% in the rural areas with a pooled prevalence of 27.4%; and 15.5% to 59.2% in the urban areas with a pooled prevalence of 33.9%. The odd of hypertension were lower in rural compared to urban dwellers [OR 0.74, 95% CI: 0.66-0.83; *p* < 0.001]. The pooled prevalence of hypertension was 32.6% in males, and 30.0% in females, with no significant difference in the odds of hypertension between the sexes [OR 0.91, 95% CI: 0.8-1.05, p = 0.196]. Comprehensive hypertension control policies are needed for both rural, and urban areas in West Africa, and for both sexes.

## Introduction

The World Health Organisation’s (WHO) African region is reported to have the highest prevalence of hypertension in the world at 46%, and it is expected to continue rising [1]. Hypertension, a condition that is both preventable and treatable, is a major contributor to the high burden of cardiovascular diseases (CVDs), mainly stroke, ischaemic heart disease, heart failure, and kidney disease, in Africa [2, 3]. In addition, CVD deaths occur at least a decade earlier in Africans compared to other populations [1, 4]. Hypertension and its complications contribute significantly to poverty in the region through catastrophic health expenditure and loss of productivity [1].

Urbanisation has been proposed as one of the main drivers of the increasing burden of hypertension and CVDs in low- and middle-income countries including those of sub-Saharan Africa (SSA) [5]. Although there is no universally accepted, or applicable definition for urbanisation; the phenomenon often reflects changes in political, social and economic forces, leading to considerable changes in lifestyle; such as changes in sources of livelihood, food, transportation, family structure, and environmental exposures [6]. Rapid urbanisation—especially if unplanned, or poorly planned as may often be the case in much of West Africa – is thought to promote development of hypertension through exposure to an environment that encourages poorer feeding habits, sedentary lifestyle, tobacco and alcohol consumption, development of obesity, and exposure to more psychological stressors [7, 8].

**Fig. 1:**
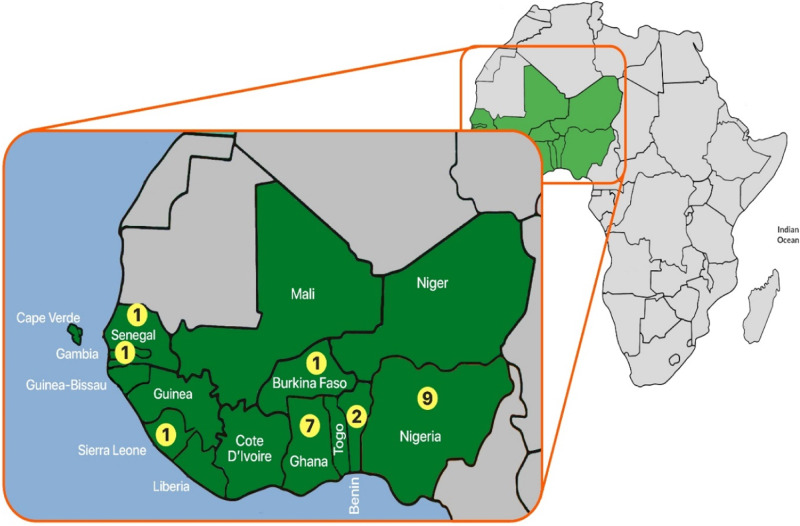
Map of Africa showing West Africa with ECOWAS member states in highlights. Insert showing the number of included studies from individual countries.

In West Africa, rural areas are generally characterised by a more active lifestyle with farming as the major occupation, a diet that contains less processed foods, and less exposure to environmental pollution compared to urban areas. However, urban populations tend to have better access to healthcare facilities, formal employment opportunities and social amenities. In addition, the population is growing faster than the amenities in many cities and towns, resulting in the creation of urban slums which are overcrowded and associated with poor living conditions [6, 8]. The urban population of West Africa is reported to have grown by 40 million between the years 2000 and 2010 [6]. At present, 15 countries in the region comprise a regional bloc called the Economic Community of West African States (ECOWAS) (Fig. 1) whose health body, the West African Health Organisation (WAHO), recently adopted a Regional Strategic Plan for Noncommunicable Disease Control in recognition of the morbidity and mortality caused by CVDs and other noncommunicable diseases [9].

The evidence regarding differences in the prevalence of hypertension between rural and urban areas of West Africa has been mixed, with some studies reporting higher prevalence in urban areas [7, 10, 11], some reporting higher prevalence in rural areas [12, 13], and others reporting no significant difference [14, 15]. Although there are a few previously published systematic reviews on hypertension in adults in SSA [16–18], alongside a number of review articles on the same subject [19, 20], none has specifically focused on examining the rural-urban differences in the prevalence of hypertension on the continent, or in the West African sub-region. In addition, Iwelunmor et al., conducted a scoping review on the topic of hypertension in West Africa in 2014 [21]. However, they did not perform a systematic search of the literature, nor did they explore rural-urban differences in the prevalence of hypertension in depth. Therefore, a systematic review of the existing literature is required to summarise the findings and examine if there is a difference in the prevalence of hypertension between rural and urban areas of West Africa. In addition, although hypertension is generally more prevalent in males, some studies have reported similar or even higher prevalence in females in the region [10, 13, 22, 23].

## Methodology

### Study protocol

A study protocol was developed and registered with the international prospective register of systematic reviews (PROSPERO) (registration number CRD42018087282). This study–from development of the protocol, PROSPERO registration, and conducting the review–was conducted in accordance with the Preferred Reporting Items for Systematic Reviews and Meta-Analyses (PRISMA) guidelines to ensure rigour and minimise the risk of bias [24].

### Search strategy and selection criteria

A search strategy including the key words “prevalence”, “hypertension”, “rural”, “urban” and “West Africa” in various combinations was used to search four major electronic databases: PubMed, Embase, African Journals Online (AJOL), and the WHO’s African Index Medicus (AIM). Searching the latter two databases aimed to improve access to papers published in African journals. PubMed was chosen over MEDLINE in order to access journals not indexed in the latter. The search strategy was developed after a preliminary literature search, as well as by searching PubMed’s medical subject heading’s (MeSH) browser; it contained both free text and controlled vocabulary like MeSH terms and was adapted for use across the different databases (see S1). Boolean operators and truncation were also used with the aim of making the strategy sensitive enough to retrieve as many relevant papers as possible. Reference lists of eligible studies as well as those of previous reviews were also searched to identify potentially relevant studies that may have been missed by the electronic search.

**Fig. 2:**
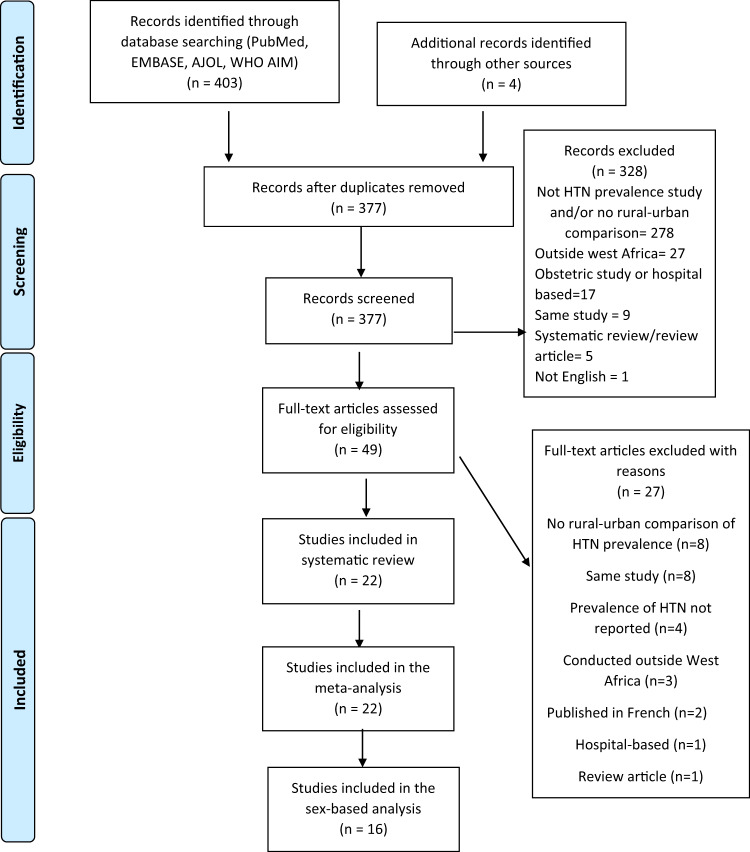
PRISMA Flow diagram of study selection. Number of records excluded at each stage with reasons provided. HTN hypertension, AJOL African Journals Online, WHO AIM World Health Organisation’s African Index Medicus.

Study selection, as well as all subsequent steps of the review, was carried out by two reviewers independently according to PRISMA guidelines. Disagreements were resolved through discussion, and the involvement of a third reviewer when necessary. Only original studies published in English language between the years 2000 and 2021 were included. This is because urbanisation in West Africa is occurring rapidly, and locations that were designated “rural” in older studies may have been reclassified [6]. Study selection was conducted in two stages. Titles and abstracts were screened and those that were not relevant to the review’s aim and objectives were excluded (Fig. 2). Full texts of potentially relevant studies were then retrieved and screened against the eligibility criteria to select the papers included in the review. Other inclusion criteria were: (i) Study was conducted in any of the West African countries (ECOWAS member states—Benin, Burkina Faso, Cape Verde, Cote d’Ivoire, Gambia, Ghana, Guinea, Guinea-Bissau, Liberia, Mali, Niger, Nigeria, Senegal, Sierra Leone, Togo), (ii) Study reports and contrasts the prevalence of hypertension between rural and urban areas of a country/countries, (iii) Study participants were adults (aged 18 years and above).

Studies were excluded based on the following: (i) Study was conducted outside of West Africa; in African-American, Afro-Caribbean, or non-black African populations, (ii) Study participants were children, (iii) Study reports the prevalence of hypertension in rural area(s) only or urban area(s) only without describing the difference between these areas, (iv) Study was published in languages other than English, (v) Obstetric study on pregnancy-induced hypertension or other hypertensive disorders in pregnancy, (vi) Hospital-based study.

### Study quality

The methodological quality of included studies was independently assessed by two reviewers using the Newcastle-Ottawa Scale (NOS) for quality assessment of non-randomised studies in systematic reviews and meta-analyses [25]. A modification of the scale for cross-sectional studies used in a previously published systematic review on pan-ethnic differences in hypertension in Europe was adapted for this review [26]. While the methodological quality of included studies was assessed, no study was excluded on these grounds.

### Data extraction

Relevant information was extracted from included studies using a pre-piloted form by two reviewers (see S2). This included: first author and year of publication; study country; study population, participant demographics, and baseline characteristics; the prevalence of hypertension in the rural and the urban locations included in each study; statistical analysis of the observed difference in the prevalence of hypertension between the rural and urban area or lack thereof, differences in the prevalence of hypertension based on sex and other factors (e.g., socioeconomic status) if reported; other cardiovascular risk factors reported; definitions for rural and urban locations as well as definitions of hypertension used; and information for the assessment of risk of bias (see S7).

### Statistical analysis

Heterogeneity between studies was assessed using the I^2^ statistic. A meta-analysis was performed on the prevalence data using a random effects model due to significant heterogeneity between the included studies to arrive at a pooled odds ratio (OR) for the rural areas, using the urban areas as the referent [25]. Pooled prevalence for rural and urban locations were also derived, although no age-standardisation was done. For studies that had a “semi-urban” category (*n* = 4) this was merged with the “urban” category in the meta-analysis. Other caveats for the meta-analysis are available in supplementary material S8. We carried out a sensitivity analysis to explore the effect of excluding the semi-urban category on the pooled estimate. Sex-based difference in the prevalence of hypertension was also explored. Those studies that presented sex-disaggregated data (*n* = 16) were included in a random effects model after testing for heterogeneity using the I^2^ statistic. We assessed publication bias using a contour-enhanced funnel plot. All statistical analyses were carried out using R version 4.02.

## Results

### General characteristics of included studies

Included studies came from seven of the fifteen countries that make up the ECOWAS with a total number of 62,907 participants. Nigeria had the largest number of studies (*n* = 9), followed by Ghana (*n* = 7) and Benin (*n* = 2), while Burkina Faso, the Gambia, Senegal and Sierra Leone contributed one study each (Fig. 1). Four of the represented countries are Francophone, while the remainder are Anglophone countries. Sample size ranged from 422 to 9,367, with large variations in response rate (range 53.4% to 100%), although not all studies reported it. The proportion of female participants ranged from 0% to 100%, while that of rural participants ranged from 31.7% to 77.8%. Study population differed widely between studies with some including only older adults or the elderly, while others restricted participants to only those in their 3^rd^ to 7^th^ decades of life (Table 1). Among studies that reported a mean age, this ranged from around 35 to 56 years, while one study reported a mean age above 70 years. Although two studies included participants aged 15 years and above, they were not excluded because in one of them the mean age was 35.4 years, while in the other over 80% of participants were aged above 20 years. One study had exclusively male participants [27], while another had exclusively female participants (Table 1) [28].

**Table 1:** General characteristics of included studies.

s/no	First author, Year of publication, Country	Study population	Sample size (*N*) {Response rate}	Rural participants *N* (%)	Female participants *N* (%)	Mean age ± SD (years)	Overall prevalence of HTN (%)	Prevalence of HTN in rural area(s) (%)	Prevalence of HTN in urban area(s) (%)
1.	Abegunde, 2013, Nigeria [14]	Elderly persons (aged ≥ 60 years) residing in Oyo state, South-western Nigeria	630 {98.4%}	314 (49.8)	385 (61.1)	[NR] (range 60– 110 years) Rural 70.8 ± 8.1 Urban 72.2 ± 9.5	36.5	34.7	38.3
2.	Agyemang, 2006, Ghana [12]	Residents of Ashanti region of Ghana	1431 {Reported as “high”}	578 (40.4)	787 (55.0)	35.9 ± 0.8	29.4	[NR] Males 27.0 Females 27.0	[NR] Males 33.4 Females 28.9
3.	Agyemang, 2017, Ghana [31]	Adults aged 25–70 years living in rural and urban Ghana	2492 {NR}	1034 (41.9)	1671 (67.1)	[NR] Rural men 46.2 Rural women 46.7 Urban men 46.5 Urban women 44.7	[NR]	[NR] Men 22.2 Women 27.9	[NR] Men 34.2 Women 29.4
4.	Banigbe, 2020, Nigeria [27]	Male partners (aged ≥ 18 years) of pregnant women participating in a “Healthy Beginning Initiative” between 2016–2018 in Benue State, North-central Nigeria	6538 {NR}	5087 (77.8) Semi-urban 1204 (18.4)	0 (0)	[NR] Median age 31 years (IQR: 26 – 37 years)	23.4	23.2 Semi-urban: 21.8	34.8
5.	Cappuccio, 2004, Ghana** [37]	Residents of Ashanti region of Ghana aged 40–75 years	1013 {53.4%}	481 (47.5)	628 (62.0)	54.7 ± 11.3	28.7	24.1	Semi-urban 32.9
6.	Ejim, 2013, Nigeria [15]	Residents of Enugu state, South-eastern Nigeria aged 40–70 years	543 {NR} Rural 70.4% Urban 40%	308 (56.7)	373 (68.7)	56.3 ± 9.9	47.7	45.1	51.1
7.	Houehanou, 2015, Benin [10]	Randomly selected national sample of persons in Benin aged above 24 years and below 65 years.	6762 {99%}	4491 (66.4)	3345 (49.5)	42.8 ± 0.3	28.4	27.5	29.9
8.	Kodaman, 2016, Ghana [7]	Residents of Brong Ahafo region of Ghana aged ≥18 years	3317 {NR}	1052 (31.7)	1876 (56.6)	[NR] (range 18 – 99 years) Rural Males 44.9 ± 17.2 Females 43.9 ± 15.9 Urban Males 42.9 ± 12.6 Females 42.1 ± 11.3	[NR]	[NR] Males 20 Females 21	[NR] Males 34 Females 32
9.	Minicuci, 2014, Ghana [11]	Nationally representative sample of ageing population (aged 50 years and above) across the 10 regions of the country	4724 {95.9%}	2806 (59.4)	2376 (50.3)	[NR] (modal age group 50 – 64 years)	Self-reported HTN: 14.2 Measured HTN: 51.1	Self-reported HTN: 8.0 Measured HTN: 45.6	Self-reported HTN: 23.1 Measured HTN: 59.2
10.	Ntandou, 2009, Benin [13]	Persons aged 25–60 years who had been living in the study area for at least 6 months	541 {NR}	170 (31.4) Semi-urban 171 (31.6)	270 (49.9)	[NR] Men 37.3 ± 10.1 Women 39.0 ± 10.0	[NR]	24.1	semi-urban 21.6 urban 26.5
11.	Obirikorang, 2015, Ghana [33]	Residents of Ashanti region of Ghana	672 {NR}	360 (53.6)	360 (53.6)	[NR] (Median age 50 years (IQR) 39–58)	34.8	36.7	32.7
12.	Odili, 2020, Nigeria [30]	Adults (aged ≥ 18 years) from 12 rural and urban communities from a state each in the 6 geopolitical zones in Nigeria.	2503 {77.3%}	1332 (53.2)	1424 (56.9)	43.8 ± 16.2	32.0	[NR] South-East 60 South-South 46.5 South-West 41.3 North-Central 18.0 North-West 27.2 North-East 25.6	[NR] South-East 53.6 South-South 37.7 South-West 39.4 North-Central 20.7 North-West 15.6 North-East 35.1
13.	Odland, 2020, Sierra Leone [23]	Individual over 40 years randomly selected in a household survey based on WHO STEPS survey	2071 {NR}	1302 (62.9)	1015 (49.0)	[NR] Median age 51.0 years (IQR 45.0 to 63.0 years)	49.6	46.0	55.8
14.	Ogah, 2013, Nigeria [50]	Residents of Abia state, South-Eastern Nigeria aged ≥ 18 years	2928 {99.5%}	1553 (53.0)	1528 (52.2)	41.8 ± 18.5	[NR] Systolic HTN 31.4 Diastolic HTN 22.5	[NR] Systolic HTN Men 33.5 Women 30.5 Diastolic HTN Men 23.4 Women 25.4	[NR] Systolic HTN Men 33.6 Women 26.4 Diastolic HTN Men 20.6 Women 18.4
15.	Oguoma, 2015, Nigeria [51]	Residents of Delta and Lagos states aged ≥ 18 years	422 {NR}	326 (77.2)	273 (64.7)	[NR] Females 42.9 ± 20.7 Males 38.3 ± 20.5	35.7	[NR] Abbi 37.3 Kwale 23.3	53.3
16.	Okello, 2020,Nigeria** [36]	Adults aged ≥18 years from seven countries in East and West Africa. Included 3 rural and 1 “semi-urban” sites in Nigeria	2065 from Nigeria {100%}	1576 (76.3) Semi-urban 489 (23.7)	1162 (56.3)	N/A^ Rural Ogane-Uge: 39.2 ± 19.5 Okpok Ikpa: 38.5 ± 14.0 Olorunda Abaa: 41.2 ± 12.9 Semi-urban Ikire: 48.1 ± 18.1	N/A^	[NR] Ogane-Uge: 33.0 Okpok Ikpak: 20.4 Olorunda Abaa: 23.3	Semi-urban Ikire: 38.6
17.	Okpechi, 2013, Nigeria [32]	Residents of Abia state, South-eastern Nigeria aged ≥18 years	2983 {99.5%}	1587 (53.2)	1553 (52.1)	41.7 (SEM = ± 0.3)	31.4	32.0	30.7
18.	Oyekale, 2019, Ghana [28]	Women aged 15-49 years in a nationwide Demographic and Health Survey (DHS) in 2014	9367 {97%}	4329 (46.2)	9367 (100)	[NR] Age range 15-49 years.	13.3	9.7	16.3
19.	Seck, 2014, Senegal [34]	Residents of St Louis, Senegal aged ≥18 years living in the study area for at least 3 months	1036 {99%}	458 (44.2)	622 (60)	48.0 ± 16.9	39.1	33.8	43.3
20.	Soubeiga, 2017, Burkina Faso [29]	Nationally representative sample of persons aged 25 to 64 years who had been residing in the country for at least six months on the day of the survey	4629 {96.4%}	3600 (77.8)	2399 (51.8)	[NR] (modal age group 25 – 34 years)	18	15.4	24.8
21.	Umuerri, 2020, Nigeria [52]	Adults aged ≥18 years from two communities in Delta State who have lived in the study sites for at least one (1) year	852 {NR}	377 (44.2)	476 (55.9)	42.6 ± 16.1	29.3	21.8	35.4
22.	van der Sande, 2000, Gambia [48]	Persons aged ≥ 15 years who had been residing in the country for at least six months on the day of the survey	5389 {78.1%}	3223 (59.8)	3161 (58.6)	35.4 (±NR)	18.4	17.8	20.3

**rural and semi-urban participants only. No urban participants. ^includes data from outside west Africa.

[*NR*] = Not reported; *N/A* = not applicable; *BP* = blood pressure; *SBP* = systolic blood pressure; *DBP* = diastolic blood pressure; *HTN* = hypertension; *SD* = Standard deviation; *SEM* = standard error of means.

Seven studies included what was reported as a nationally representative sample; or reported having study sites from all geopolitical regions of the study country [10, 11, 23, 28–31], while others were conducted only within a particular state or region of the study country. Seven of the included studies [10, 11, 23, 29, 32–34] used the WHO STEPwise approach, a framework of standardised research tools developed by the WHO for surveillance of NCDs and NCD risk factors [35]. The majority of studies compared rural and urban locations only, with a total of 36,353 and 24,158 participants from these locations respectively; while four studies had a semi-urban location which contributed a total of 2,396 participants [13, 27, 36, 37]. In two of these studies, the semi-urban location was in addition to the rural and urban locations [13, 27], while in the remaining two, there was no urban location [36, 37].

### Prevalence of hypertension

The reported overall prevalence of hypertension varied from 13.3% in a study of mostly young women to 51.1% in a study of persons aged ≥50 years [11, 28]. The prevalence of hypertension in rural and urban areas differed broadly between studies with a pooled prevalence of 27.4% [95% CI: 23.5–31.7%] and 33.9% [95% CI: 29.3–38.7%] respectively, although no age age-standardisation was done. The lowest reported rural prevalence of hypertension was 9.7% in Ghana, and the highest was 60% in South-East Nigeria (Table 1) [28, 30]. While for the urban areas, the reported prevalence ranged from 15.5% to 59.2%. The prevalence of hypertension in males ranged from 19.4% to 51.5% while in females it ranged from 13.3–54.8% [11, 23, 28, 29].

### Rural-urban difference in the prevalence of hypertension

The majority of studies (19) reported the overall prevalence of hypertension to be higher in urban than in rural areas, with 13 of them reporting the difference to be statistically significant. Among the remaining 3 studies only one reported a statistically significant higher prevalence of hypertension in the rural compared to urban areas.

### Meta-analysis

**Fig. 3:**
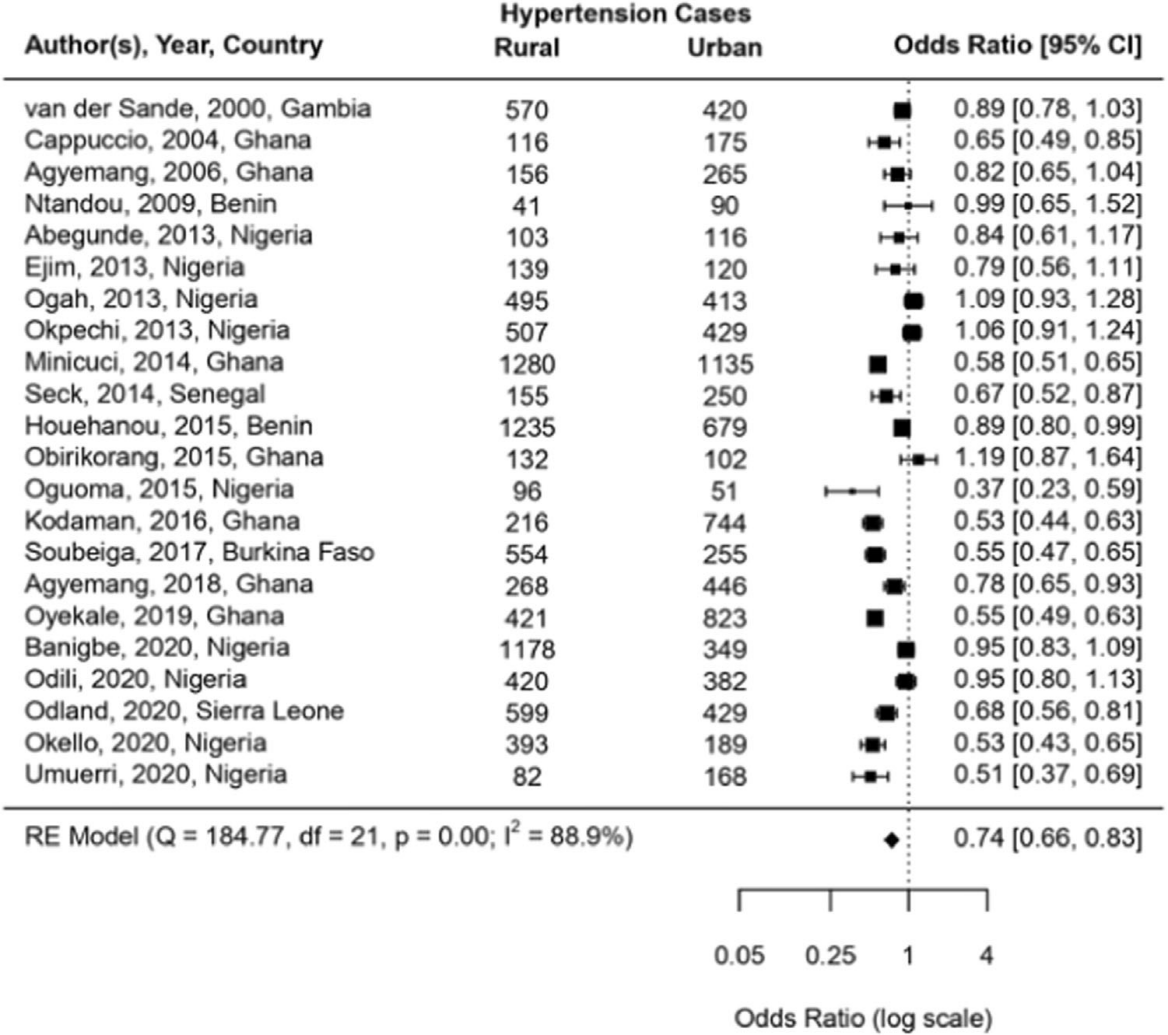
Rural-urban difference in the prevalence of hypertension in West Africa. Results from the random effects meta-analysis model indicate a lower odd of hypertension among persons living in rural compared to urban areas.

**Fig. 4:**
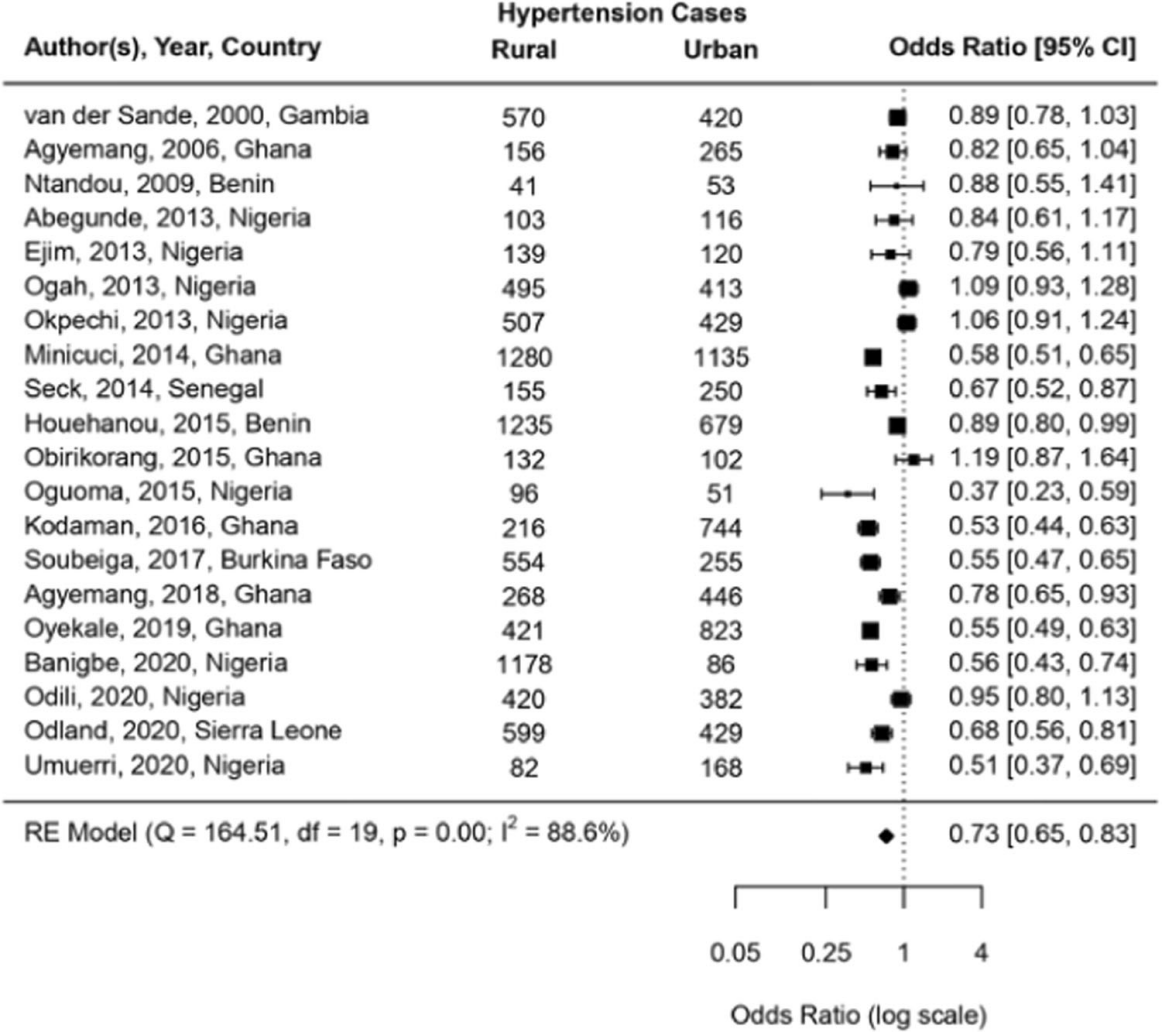
Rural-urban difference in the prevalence of hypertension in West Africa after excluding persons living in semi-urban areas. Significantly lower odds of hypertension in persons living in rural compared to urban areas still present after excluding persons living in semi-urban areas.

**Fig. 5:**
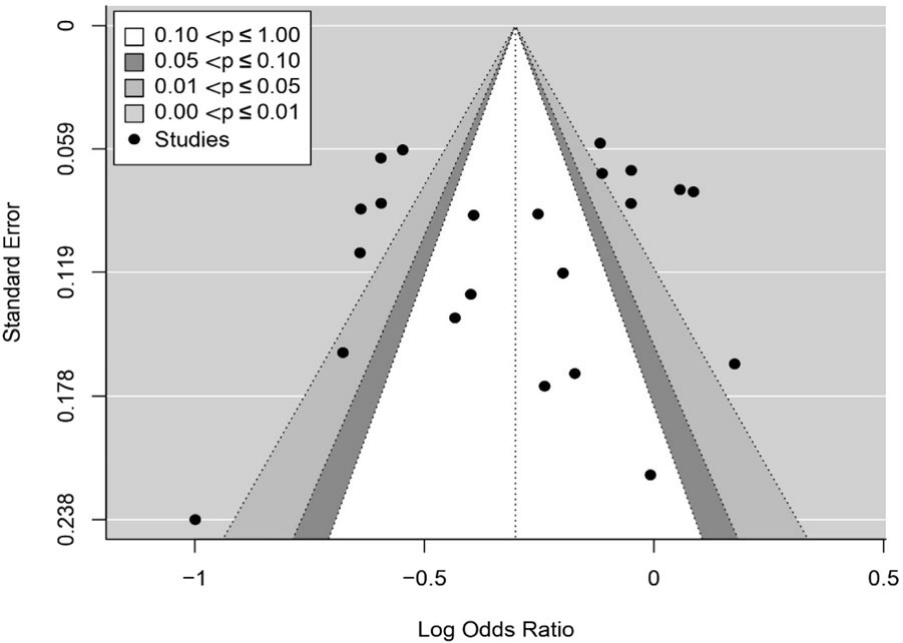
Contour-enhanced funnel plot for evaluating bias and heterogeneity among the included studies. Plot was statistically symmetrical over the pooled odds ratio estimate.

**Fig. 6:**
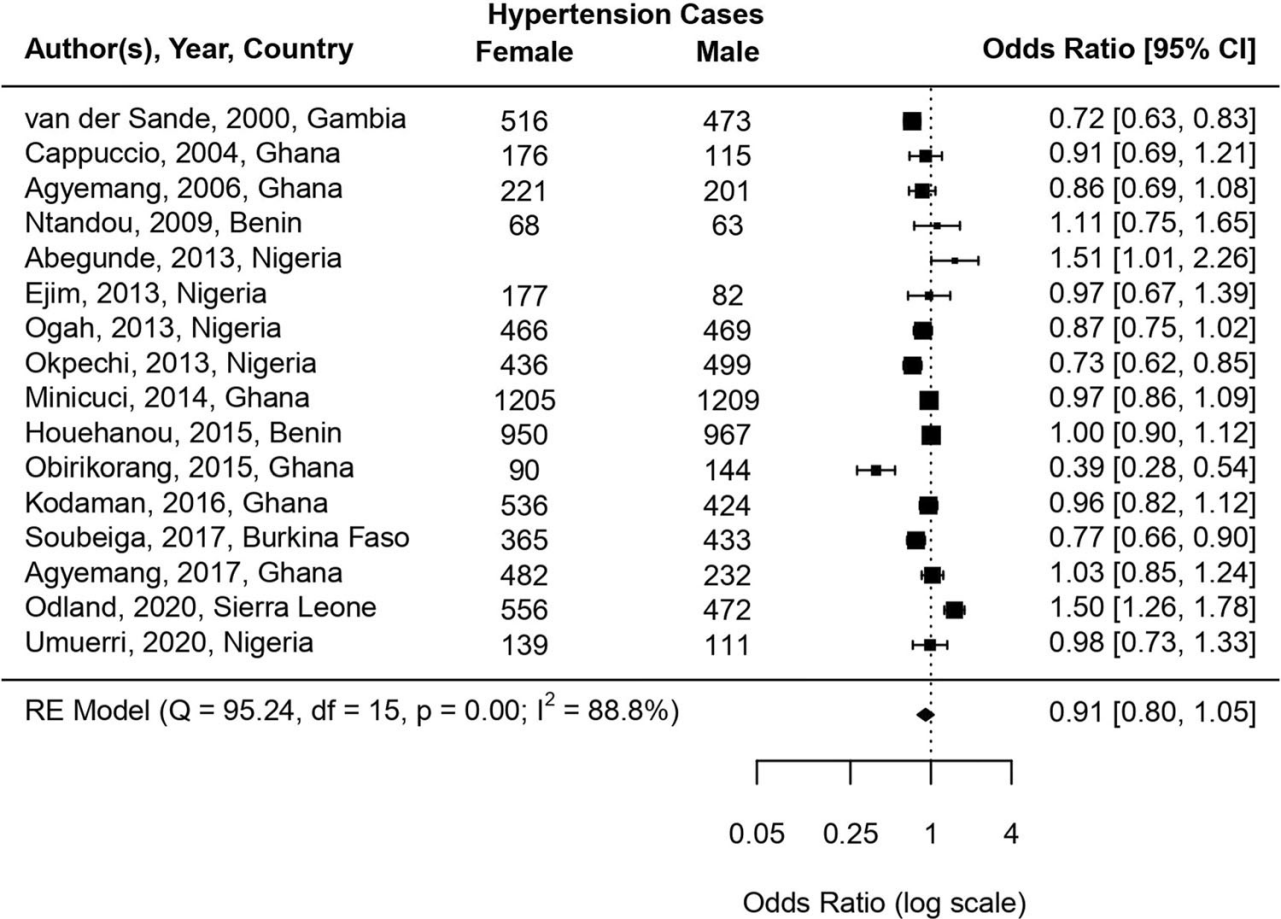
Sex-based analysis of prevalence of hypertension in West Africa with males as the referent. No significant difference in the odds of hypertension between males and females found.

All 22 studies were included in a meta-analysis. Heterogeneity was significant with an I^2^ of 88.9%, thus a random effects model was used to estimate the OR for rural compared to urban areas. The results of the meta-analysis indicate that the odds of hypertension were lower among rural residents compared to their urban counterparts [OR = 0.74, 95% CI: 0.66–0.83; *p* < 0.001] (Fig. 3). The results remained similar after the sensitivity analysis in which we excluded participants living in semi-urban areas [OR = 0.73, 95% CI: 0.65–0.83; *p* < 0.001] (Fig. 4). Publication bias was evaluated using a contour-enhanced funnel plot. Both the Egger Test (z = -0.66, *p* = 0.50) and the rank correlation test of funnel plot asymmetry (Kendall’s tau = -0.05, *p* = 0.78) suggest that the funnel plot was statistically symmetrical over the pooled OR estimate (Fig. 5). Females had lower odds of hypertension compared to males, but the difference was not statistically significant [OR 0.91, 95% CI: 0.8–1.05, *p* = 0.196] (Fig. 6).

### Quality of included studies

A modified Newcastle-Ottawa scale was used to assess the quality of included studies, with the major parameters assessed being selection bias, exposure and outcome ascertainment, statistical analysis, and adjustment for confounders (supplementary material S5, sample form). Out of a possible maximum score of 10, the lowest score was 3, while the highest was 9. All included studies selected a sample that was either truly or somewhat representative of the target population, with nearly all employing a random sampling technique. Although the majority of the included studies gave a response rate, most studies lost the score for description of non-responders. A number of studies classified rural and urban locations using parameters specified by the study country’s statistical or population body, while some did not specify which parameters were used to classify these locations. No study relied solely on self-report of hypertension, all studies measured blood pressure to define hypertension (supplementary material S6, full quality assessment table).

## Discussion

This systematic review and meta-analysis of 22 studies examined the difference in the prevalence of hypertension between rural and urban areas of West Africa. We observed that likelihood of hypertension was 26% lower among people living in rural areas compared to those living in urban areas. We also explored the sex differences in the prevalence in hypertension whereby the likelihood of hypertension was comparable between males and females.

The prevalence of hypertension varied widely between the included studies, a finding consistent with that of previously published reviews of hypertension in SSA and West Africa; but generally increased with the age of the population studied [16–18, 38]. The prevalence of hypertension in rural and urban areas also varied significantly between studies, with a pooled prevalence of 27.4% and 33.9% for rural and urban areas respectively, although no age-standardisation was done before pooling the prevalence. The majority of the included studies reported a statistically significant higher prevalence in urban compared to the rural areas. Furthermore, the pooled odds of hypertension were 26% lower in rural compared to urban residents. The lower prevalence of hypertension in rural compared to urban areas is similar to findings from a recently published systematic review and meta-analysis of hypertension in Nigeria by Adeloye et al. where the pooled prevalence in rural and urban areas was 25.5% (95% CI: 21.1– 29.9%) and 33.5% (95% CI: 25.1–42.0%) respectively [39]. Similarly, in the systematic review of studies on hypertension in Ghana by Bosu, those studies that had both rural and urban sites consistently reported higher prevalence in the urban sites, although no meta-analysis was performed [22]. The similarity in findings between these and our review may be partly because the majority of studies in our review were from Nigeria and Ghana. In contrast, some other reviews on hypertension in Africa have reported comparable prevalence between rural and urban areas [17, 18]. A meta-analysis of studies on hypertension in Africa reported a significantly higher prevalence in urban compared to rural areas in the year 1990 but no difference between these locations in 2000 and 2010 [17].

Potential reasons for a higher prevalence of hypertension in urban areas include socioeconomic and lifestyle changes that may lead to higher prevalence of its risk factors like overweight, obesity, smoking, and diabetes mellitus [40]. In our review, the prevalence of diabetes mellitus and obesity were reported to be higher among urban residents in many of the included studies that reported on these factors. A recent meta-analysis on obesity in Nigeria reported a similar finding [41]. Dietary factors, especially consumption of processed high-salt containing foods may also be a contributor [39, 41]. Other mechanisms are thought to involve psychosocial stress in urban residents resulting from financial stress, redefinition of cultural identity, and movement away from traditional coping mechanisms, including social support from extended family [21]. Air pollution which tends to be worse in urban areas due to exhaust fumes from motorised vehicles and industries may also be contributory [3]. Some researchers have even suggested an association between duration of urban residence and risk of hypertension in the region [42, 43]. Although conducted in East Africa, the Kenyan Luo migration study has demonstrated a rise in BP in a cohort migrating from a rural to an urban area and followed up for two years, compared to controls residing in the rural area [44]. However, better access to healthcare facilities in urban compared to rural areas may also contribute to a higher reported prevalence in these areas [40].

A limited analysis of 16 studies suggests no significant difference in the prevalence of hypertension between males and females in West Africa, although there was a trend towards a higher prevalence in males. This finding has been reported in other reviews on hypertension in the region. For instance, in a systematic review of studies on hypertension in Ghana, Bosu observed no difference in the prevalence of hypertension between males and females [22]. Similarly, a meta-analysis on hypertension in Africa by Adeloye and colleagues reported no significant difference in the prevalence of hypertension between males and females the years 2000 and 2010, although they observed a higher prevalence in males compared to females in the year 1990 [17]. Other reviews have reported a significantly higher prevalence in males, although Akinlua et al. included papers from as far back as 1968 and thus, may not be representative of the current situation [38, 45].

Traditionally, hypertension is reported to be more prevalent in males compared to pre-menopausal females, chiefly due to the effect of protective hormones in the latter [46]. The absence of a significant difference in the odds of hypertension between males and females observed in this review may have been influenced by the age of participants across studies in our review with the mean age of participants being clustered around late thirties to fifties, and a number of studies being exclusively in older persons. Consequently, some of the females may have been peri- or post-menopausal, at which time their hormonal profile is no longer protective of hypertension and CVDs [46]. Other possible contributory factors to this finding include lower level of physical activity, higher prevalence of overweight/obesity, and a generally higher body mass index in females compared to males in West Africa, a finding reported by some studies in our review and supported by a recent review and meta-analysis of obesity in Nigeria [41]. Yet other potential mechanisms may involve women suffering a psychosocial and emotional impact of insecurity, and civic unrest, as well as socioeconomic hardships in the region [39].

### Strengths and limitations

A major strength of this review is that all steps were carried out by at least two reviewers according to the transparently described pre-specified protocol in accordance with PRISMA guidelines. The literature search likely retrieved the vast majority of relevant studies since the search strategy was developed after thorough literature scoping; and two of the electronic databases searched are the largest for biomedical publications while the remaining two index papers published in African journals or about Africa/Africans [47]. None of the studies that met the inclusion criteria were excluded on the grounds of quality, as has been done in previously published reviews, and a thorough quality assessment was done [17]. A universal strength of all included studies was that the outcome (i.e., hypertension) was assessed by measuring the blood pressure of all participants, with many of the studies taking several precautions to ensure accurate measurement.

This review is not without its limitations. The lack of a standardised definition for rural and urban locations among included studies could have introduced bias and makes the detected rural-urban difference difficult to interpret, especially with many of the studies not reporting what parameters were employed to classify locations. However, this is oftentimes the case with studies on rural-urban comparisons [6]. Few studies specified minimum duration of residency in the study location for inclusion into the study [13, 29, 34, 48]. In addition, it is not uncommon in many African countries for people to work in cities but reside in surrounding smaller towns and villages, partly due to higher cost of living [49]. These factors could lead to misclassification of participants; and make interpretation of the relationship between rural and urban residency and the development of hypertension as was found in this review, complex. Although a random effects meta-analysis was carried out due to high heterogeneity, caution is advised when interpreting the results as the detected difference in the odds of hypertension could be partly attributable to the inherent differences between the included studies. Another limitation of the meta-analysis is lack of age-standardisation of the prevalence of hypertension. Finally, the sex-based meta-analysis was carried out for only a sub-set of papers. Therefore, the findings may not be fully representative of the whole population.

### Implications for research and policy

Although some West African countries, notably Benin, Burkina Faso, Ghana and Sierra Leone have generated recent large-scale nationwide data on hypertension [10, 11, 23, 29], many others have not. There is a need for all countries in the region to generate updated comprehensive data on the prevalence, treatment, control and risk factors for hypertension, and indeed other recognised CVD risk factors. This will allow for better understanding of the problem and could help inform development of policies to tackle it. A wider adoption of the WHO STEPS instrument will allow for better comparison of hypertension and CVD data within and between countries, regions of the continent; and the world at large. The West African Health Organisation (WAHO), the health organ of the ECOWAS, could facilitate this by offering central coordination and fostering collaboration between the countries.

A uniform definition of rural and urban settings among researchers in the region is timely, since social conditions are somewhat similar, and definitions from more developed parts of the continent or other regions of the world may not be applicable or appropriate. The definitions proposed by Moriconi-Ebrard et al., or Benin Republic’s Statistical and Economic Analysis Institute (INSAE) may be good starting points [6, 10].

With the high burden of hypertension in both rural and urban areas of the region, comprehensive control measures are urgently needed to curtail this epidemic. Additional research into factors contributing to a potentially higher prevalence among urban dwellers is also necessary, in order to factor some of these into urban planning. Moreover, studies aimed at investigating the rural-urban difference in hypertension in the region need to be improved. We propose the following improvements to such studies: (1) adoption of a uniform definition for rural and urban locations, and categories between these two extremes (e.g., semi-urban) by researchers in the region; (2) enroling larger sample sizes, preferably nationally representative samples from rural and urban locations across all geopolitical regions of the study country; (3) using a uniform standardised definition for hypertension and standardised blood pressure measurement; and (4) presenting data disaggregated by sex in a manner to allow exploration of the rural-urban differences by sex. Lastly, the need to investigate the reasons for the narrowing gap in hypertension prevalence between males and females with the aim of instituting measures to arrest or reverse this trend cannot be overemphasised.

Although this review is not without its imitations as highlighted above, and the socioeconomic context of West Africa may differ from other regions of the continent; we believe its findings, especially as regards the rural-urban difference, may be applicable to other regions of SSA.

### Summary table

#### What is known about the topic

- Africa has the highest prevalence of hypertension of all the WHO regions. Hypertension prevalence in West Africa is rising and urbanisation is thought to be one of the drivers.
- Studies comparing hypertension prevalence in between urban and rural areas of West Africa have had mixed results.

#### What this study adds

- This systematic review and meta-analysis of the literature found high prevalence of hypertension in both rural and urban areas of West Africa with lower odds among urban dwellers.
- Limited sex analysis revealed no significant difference in the prevalence of hypertension between males and females.
- This study identified the need for a uniform definition for rural and urban (and semi-urban settings) in the region to allow for better comparison between these settings.

### Supplementary information

Supplementary Material 1

Supplementary Material 2

Supplementary Material 3

Supplementary Material 4

Supplementary material 5 updated

Supplementary Material 6

Supplementary Material 7

Supplementary Material 8

## Data Availability

The data collected and analysed in this study can be found within the published article and its supplementary files. Additional data are available from the corresponding author on reasonable request.
