# Supplementary material for: Rural-urban difference in the prevalence of hypertension in West Africa: a systematic review and meta-analysis
Source: J Hum Hypertens. 2022 Apr 16;38(4):352–64. doi: 10.1038/s41371-022-00688-8 (PMC11001577; doi:10.1038/s41371-022-00688-8)
Supplement: Supplementary file 1 — Supplementary Material 1 [file 41371_2022_688_MOESM1_ESM.docx]

**S1 – Search strategy PubMed**

1. ((prevalence) OR burden))

2. (((((((hypertens*) OR blood pressure) OR high blood pressure) OR elevated blood pressure) OR elevated BP) OR high BP)

3. (((west Africa) OR Africa, western) OR Western Africa)

4. 1 and 2 and 3

5. (((urban) OR urban health) OR urban population))

6. (((rural) OR rural health) OR rural population)))

7. 5 AND 6

8. ((((rural-urban) OR rural/urban) OR urban-rural) OR urban/rural))

9. 7 OR 8

10. (("2000/01/01"[PDat] : "2021/05/31"[PDat])

11. adult[MeSH])

12. 4 AND 9 AND 10 AND 11

13. Limit 12 to (English)
