# Supplementary material for: Rural-urban difference in the prevalence of hypertension in West Africa: a systematic review and meta-analysis
Source: J Hum Hypertens. 2022 Apr 16;38(4):352–64. doi: 10.1038/s41371-022-00688-8 (PMC11001577; doi:10.1038/s41371-022-00688-8)
Supplement: Supplementary file 2 — Supplementary Material 2 [file 41371_2022_688_MOESM2_ESM.docx]

**S2 – Sample of Completed data extraction form**

| Study identification:  **Abegunde, K.A. & Owoaje, E.T., 2013**. Health problems and associated risk factors in selected urban and rural elderly population groups of South-West Nigeria. *Annals of African medicine*, 12(2), pp.90–97. | |
| --- | --- |
| Country | Nigeria  (Oyo State, southwest Nigeria) |
| Target population | Elderly (≥60 years of age) |
| Sample size (response rate) | 630 (98.4%)  Non-responders not described |
| Exposure ascertainment  (i.e definition of rural/urban location of residence) | Not explicitly stated, however, the rural community was located farther away from the largest city and the community was a predominantly farming one. |
| Definition of hypertension | (JNC VI)  SBP≥140mmHg and/or DBP ≥90mmHg  OR  Being on anti-hypertensive medication*  OR  Self-reported diagnosis of hypertension*  *analysed separately from HTN by BP measurement |
| BP measurement device | Accoson mercury shygmomanometer |
| Rural participants (%) | 49.8% |
| Female participants (%) | 61.1%  Rural: 64.0%  Urban: 58.2% |
| Age (mean) | [NR]  (range 60 – 110 years)  Rural  70.8 years ± 8.1  Urban  72.2 years ± 9.5 |
| Overall prevalence of hypertension | 36.5% |
| Prevalence of hypertension in the rural area | 34.7% |
| Prevalence of hypertension in the urban area | 38.3% |
| Statistical analysis of difference between rural and urban area | X^2^ = 0.840  P=0.359 |
| Confounders adjusted for | NA |
| Factors reported to affect risk of HTN/  Other CVD risk factors reported to have significant rural/urban difference | Multivariate logistic regression shows that  Female sex (OR = 1.551; CI=1.008-2.387; P = 0.046)  Obesity (OR = 2.8; 95% CI = 1.520-5.287; P = 0.001) were associated with increasing risk of HTN.  Increasing income was associated with decreased risk of HTN;  0.798 per unit increase in naira (OR = 0.798; 95%CI = 0.677-0.940; P = 0.007).  Use of smokeless tobacco and alcohol more common in rural areas (*P*<0.007 and *P*=0.023 respectively) |
| Conclusion/Comments | Prevalence of HTN slightly higher in urban than rural areas but the difference is NOT statistically significant |

NA= Not applicable. [NR]=Not reported. BP= Blood pressure; OR= Odds ratio; CVD= cardiovascular disease; HTN=Hypertension
