# Supplementary material for: Rural-urban difference in the prevalence of hypertension in West Africa: a systematic review and meta-analysis
Source: J Hum Hypertens. 2022 Apr 16;38(4):352–64. doi: 10.1038/s41371-022-00688-8 (PMC11001577; doi:10.1038/s41371-022-00688-8)
Supplement: Supplementary file 3 — Supplementary Material 3 [file 41371_2022_688_MOESM3_ESM.docx]

**S3 – Table showing studies that reported ORs and adjusted for potential confounders using regression models**

| **Author (year)** | **Effect estimate i.e. Adjusted Odds ratio (95% CI)** | **Potential Confounders adjusted for** |
| --- | --- | --- |
| Agyemang (2006) | Urban* 2.0 (1.5 – 2.6)  Urban Males~ 1.7 (1.1 – 2.7)  Females~ 1.5 (1.0 – 2.4) | Age, level of education, smoking, alcohol, BMI, heart rate |
| Agyemang (2017)** | Urban men 1.37 (1.10–1.70)  Urban women 0.99 (0.85-1.16) | Age, BMI, level of education |
| Banigbe (2020) | Urban 1.6 (1.2–2.1)  Semi-urban 0.9 (0.8-1.1) | [NR] |
| Houehanou, (2015) | Urban 1.4 (1.2 – 1.6) | Age group, gender |
| Kodaman, (2016) | Urban 3.2 (2.6 – 4.0) | Age, sex |
| Minicuci, (2014) | Measured HTN. Rural 0.77 (0.61 – 0.97)  Self-reported HTN. Rural 0.53 (0.39 - 0.72) | Smoking, alcohol |
| Ntandou, (2009) | Semi-urban 0.36 (0.17 – 0.74)  Urban 0.38 (0.20 – 0.74) | Age |
| Odland (2020) | Urban 1.04 (1.01 – 1.08) | Obesity |
| van der Sande, (2000) | Urban 1.8 (0.9 – 3.5) for BP ≥ 160/95mmHg  Urban 2.0 (1.2 – 3.2) for BP ≥ 140/90mmHg | Age, sex |

** reported Prevalence ratio not odds ratio

*adjusted for age only. ~further adjusted for other factors listed. HTN=hypertension; BMI: Body mass index; BP=blood pressure
