# Supplementary material for: Rural-urban difference in the prevalence of hypertension in West Africa: a systematic review and meta-analysis
Source: J Hum Hypertens. 2022 Apr 16;38(4):352–64. doi: 10.1038/s41371-022-00688-8 (PMC11001577; doi:10.1038/s41371-022-00688-8)
Supplement: Supplementary file 4 — Supplementary Material 4 [file 41371_2022_688_MOESM4_ESM.docx]

**S4 – Table showing results of studies that did not report odds ratios**

| **Author, Year, Country** | **Hypertension definition** | **Overall prevalence of HTN** | **Prevalence of HTN in rural area(s)** | **Prevalence of HTN in urban area(s)** | **P values of test statistics** |
| --- | --- | --- | --- | --- | --- |
| Abegunde, 2013, Nigeria | SBP≥140 and/or DBP≥90mmHg  Or anti-hypertensive medication use*  Or self-reported HTN diagnosis* | 36.5% | 34.7% | 38.3% | *p=*0.359  (χ2 test) |
| Cappuccio**, 2004, Ghana | SBP≥140 and/or DBP≥90 mm Hg  Or anti-hypertensive medication use | 28.7% | 24.1% | 32.9% | *p=*0.002  (χ2 test) |
| Ejim, 2013, Nigeria | SBP≥140mmHg and/or DBP≥90mmHg  Or anti-hypertensive medication use | 47.7%  Systolic HTN 37.9%  Diastolic HTN 33.7% | 45.1%  Systolic HTN 40.3%  Diastolic HTN 27.9% | 51.1%  Systolic HTN 34.9%  Diastolic HTN 41.3% | *p=*0.193  Systolic HTN *p=*0.212  Diastolic HTN *p=*0.001  (χ2 test) |
| Obirikorang, 2015, Ghana | SBP≥140mmHg and/or DBP≥90mmHg | 34.8% | 36.7% | 32.7% | *p=*0.2921  (Fisher’s exact test) |
| Odili, 2020, Nigeria | SBP greater ≥ 140 mmHg or DBP greater ≥ 90 mmHg  Or reported use of antihypertensive medication. | 32.0% | South-East  60%  South-South  46.5%  South-West 41.3%  North-Central  18.0%  North-West  27.2%  North-East  25.6% | South-East  53.6%  South-South  37.7%  South-West  39.4%  North-Central 20.7%  North-West  15.6%  North-East  35.1% | [NR] |
| Ogah, 2013, Nigeria | SBP≥140mmHg and/ or DBP ≥ 90mmHg  Systolic HTN SBP≥140mmHg and DBP<90mmHg  Diastolic HTN SBP<140mmHg and DBP≥90mmHg | [NR]  Systolic HTN 31.4%  Diastolic HTN 22.5% | [NR]  Systolic HTN  Men 33.5%  Women 30.5%  Diastolic HTN  Men 23.4%  Women 25.4% | [NR]  Systolic HTN  Men 33.6%  Women 26.4%  Diastolic HTN  Men 20.6%  Women 18.4% | [NR]  [NR]  [NR]  [NR]  [NR] |
| Oguoma, 2015, Nigeria | SBP ≥ 130mmHg and/or DBP ≥ 85mmHg | 35.7% | [NR]  Abbi 37.3%  Kwale 23.3% | 53.3% | [NR] |
| Okello**, 2020, Nigeria | JNC 7  SBP ≥140 mmHg  and/or  DBP ≥90 mmHg  and/or  self-report of previous diagnosis with or without current treatment with antihypertensive medications | N/A^ | Ogane-Uge:  Crude prevalence 33.0% (95% CI: 28.4, 37.7)  Age-standardised  27.7% (95% CI: 24.4, 30.9)  Okpok Ikpak:  Crude prevalence  20.4% (95% CI: 17.9, 25.6)  Age-standardised 12.6% (95% CI: 11.2, 14.1)  Olorunda Abaa:  Crude prevalence  23.3% (95% CI:20.3, 26.6)  Age-standardised 20.8% (95% CI:18.4, 23.2) | Semi-urban  Ikire:  Crude prevalence  38.6 % (95% CI: 34.2, 43.0)  Age-standardised 27.5 (24.6, 30.4) | [NR] |
| Okpechi, 2013, Nigeria | SBP≥140 mmHg and or DBP≥90 mmHg  Or anti-hypertensive medication use | 31.4% | 32.0% | 30.7% | *p>*0.05  (Pearson correlation coefficient) |
| Oyekale, 2019, Ghana | “medically confirmed to have raised blood pressures that require regular intake of some medications and those for which the average values of their last two blood pressure measurements for systolic or diastolic were ≥140 mm Hg or ≥90 mm Hg, respectively. “ | 13.28% | 9.73% | 16.34% | [NR]  (Probit regression model of obesity on hypertension) |
| Seck, 2014, Senegal | SBP ≥140mmHg and/or DBP ≥90mmHg  or  any prescription of antihypertensive medication in the past 2 weeks,  or any self-reported history of hypertension. | 39.1% | 33.8% | 43.3% | *p=*0.002  (Pearson χ2 test) |
| Soubeiga, 2017, Burkina Faso | SBP≥140mmHg  and/or DBP ≥90mmHg  Or anti-hypertensive medication use | 18% | 15.37% | 24.81% | *p<* 0.001  (Fisher’s exact test) |
| Umuerri, 2020, Nigeria | JNC 7  SBP≥140mmHg  Or  DBP≥90mmHg  Or  self-reported history of HTN as diagnosed by a healthcare professional irrespective of their current BP readings or use of antihypertensive drugs | 29.3% | 21.8% | 35.4% | *p<*0.001  (χ2 test and Spearman’s rank correlation coefficient) |

^ includes data from outside west Africa. ** rural and semi-urban participants only. No urban participants.

[NR]= not reported; N/A= Not applicable; SBP= systolic blood pressure; DBP= diastolic blood pressure; HTN= hypertension
