# Supplementary material for: Rural-urban difference in the prevalence of hypertension in West Africa: a systematic review and meta-analysis
Source: J Hum Hypertens. 2022 Apr 16;38(4):352–64. doi: 10.1038/s41371-022-00688-8 (PMC11001577; doi:10.1038/s41371-022-00688-8)
Supplement: Supplementary file 5 — Supplementary material 5 updated [file 41371_2022_688_MOESM5_ESM.docx]

**S5 – Sample of completed modified Newcastle-Ottawa scale for quality assessment~**

| STUDY IDENTIFICATION | **Agyemang, C., 2006.** Rural and urban differences in blood pressure and hypertension in Ghana, West Africa. *Public health*, 120(6), pp.525–533. |  |
| --- | --- | --- |
| SELECTION (MAXIMUM 5 STARS) | 1. Representativeness of the sample: 2. Truly representative of the average in the target population (all subjects or random sampling). * 3. Somewhat representative of the target population (non-random sampling). * 4. No description of sampling strategy. | * |
|  | 1. Sample size: 2. Justified and satisfactory. * 3. Not justified. |  |
|  | 1. Non-respondents: 2. Comparability between respondents’ and non-respondents’ characteristics is established, and the rate is satisfactory. * 3. The response rate is unsatisfactory, or the comparability between respondents and non-respondents is unsatisfactory. 4. No description of the response rate or the characteristics of the responders and the non-responders. |  |
|  | 1. Ascertainment of exposure: 2. Rural-urban classification clear (i.e. rural and urban areas well defined and distinguished) and based on official country metrics (e.g. population size, infrastructure, urbanization status, etc. as described by the country’s or other official population and/or statistical body) ** 3. Rural-urban classification described but it is not clear what metrics were used or metrics used not based on official country definitions (as described by the country’s or other population and/or statistical body) * 4. Rural-urban classification not described. | * |
| COMPARABILITY (MAXIMUM 2 STARS) | 1. The subjects in different outcome groups are comparable, based on the study design or analysis. Confounding factors are controlled. 2. The study controls for the most important confounding factor (Age). * 3. The study controls for any additional confounding factors (sex, smoking, alcohol, BMI etc.). * | ** |
| OUTCOME (MAXIMUM 3 STARS) | 1. Assessment of the outcome: 2. Objective measurement of blood pressure. ** 3. Self-reported diagnosis of hypertension. * 4. No description of how outcome (hypertension) was assessed. | ** |
|  | 1. Statistical test: 2. The statistical test used to analyse the data is clearly described and appropriate, and the measurement of the association is presented, including confidence intervals and the probability level (p value). *   b) The statistical test is not appropriate, not described or incomplete. | * |
| TOTAL NUMBER OF STARS  (MAXIMUM 10 STARS) |  | 7 |

~ quality assessment using an adapted version of the Newcastle-Ottawa scale modified for cross-sectional studies.^25,26^
