# Supplementary material for: Rural-urban difference in the prevalence of hypertension in West Africa: a systematic review and meta-analysis
Source: J Hum Hypertens. 2022 Apr 16;38(4):352–64. doi: 10.1038/s41371-022-00688-8 (PMC11001577; doi:10.1038/s41371-022-00688-8)
Supplement: Supplementary file 6 — Supplementary Material 6 [file 41371_2022_688_MOESM6_ESM.docx]

**S6 - Table showing Quality Assessment of included studies**

|  | **SELECTION**  **Maximum 5 stars** | | | | **COMPARABILITY**  **Maximum 2 stars** | **OUTCOME**  **Maximum 3 stars** | | **TOTAL NO. OF STARS**  **Maximum 10 stars** |
| --- | --- | --- | --- | --- | --- | --- | --- | --- |
| First author, Year, Country | Representativeness of the sample | Sample size | Non-respondents | Ascertainment of exposure | The subjects in different outcome groups are comparable, based on the study design or analysis. Confounding factors are controlled | Assessment of outcome | Statistical test |  |
| Abegunde, 2013, Nigeria | * |  |  | * |  | ** | * | 5 |
| Agyemang., 2006, Ghana | * |  |  | * | ** | ** | * | 7 |
| Agyemang, 2017, Ghana | * | * |  |  | ** | ** | * | 7 |
| Banigbe, 2020, Nigeria | * |  |  |  | * | ** | * | 5 |
| Cappuccio, 2004, Ghana | * |  | * |  |  | ** | * | 5 |
| Ejim, 2013, Nigeria | * |  |  | * |  | ** | * | 5 |
| Houehanou, 2015, Benin | * | * |  | ** | ** | ** | * | 9 |
| Kodaman, 2016, Ghana | * |  |  | * | ** | ** | * | 7 |
| Minicuci, 2014, Ghana | * |  |  |  | * | ** | * | 5 |
| Ntandou, 2009, Benin | * |  |  | ** | * | ** | * | 7 |
| Obirikorang, 2015, Ghana | * |  |  | * |  | ** | * | 5 |
| Odili, 2020, Nigeria | * |  |  | * |  | ** |  | 4 |
| Odland, 2019, Sierra Leone | * | * |  | ** | * | ** | * | 8 |
| Ogah, 2013, Nigeria | * | * |  | ** |  | ** |  | 6 |
| Oguoma, 2015, Nigeria | * | * |  |  |  | ** |  | 4 |
| Okello, 2020, Nigeria | * |  |  |  | * | ** |  | 4 |
| Okpechi, 2013, Nigeria | * | * |  |  |  | ** | * | 5 |
| Oyekale, 2019, Ghana | * |  |  |  |  | ** |  | 3 |
| Seck, 2014, Senegal | * | * |  |  |  | ** | * | 5 |
| Soubeiga, 2017, Burkina Faso | * | * |  |  |  | ** | * | 5 |
| Umuerri, 2020, Nigeria | * | * |  | * |  | ** | * | 6 |
| Van der Sande, 2000, Gambia | * |  |  | * | ** | ** | * | 7 |
