# Supplementary material for: Rural-urban difference in the prevalence of hypertension in West Africa: a systematic review and meta-analysis
Source: J Hum Hypertens. 2022 Apr 16;38(4):352–64. doi: 10.1038/s41371-022-00688-8 (PMC11001577; doi:10.1038/s41371-022-00688-8)
Supplement: Supplementary file 7 — Supplementary Material 7 [file 41371_2022_688_MOESM7_ESM.docx]

**S7 – Expanded data extraction table for all included studies**

| **Author, Year, Country** | **Population** | **Sample size (N), {response rate}** | **Rural participants (%)** | **Sex (% female)** | **Mean age ± SD (years)** | **Overall prevalence of HTN** | **Prevalence of HTN in rural area(s)** | **Prevalence of HTN in urban area(s)** | **Exposure ascertainment** | **Outcome ascertainment** | **Statistical analysis of difference between rural and urban area** | **Potential confounders adjusted for** | **Factors reported to affect risk of HTN** | **Other CVD risk factors reported to have significant rural-urban difference** |
| --- | --- | --- | --- | --- | --- | --- | --- | --- | --- | --- | --- | --- | --- | --- |
| Abegunde, 2013, Nigeria | Elderly persons (≥60 years of age) residing in Oyo state, south western Nigeria | 630 {98.4%} | 49.8% | 61.1%  Rural  64.0%  Urban  58.2% | [NR]  (range 60 – 110 years)  Rural  70.8 ± 8.1  Urban  72.2 ± 9.5 | 36.5% | 34.7% | 38.3% | Rural area located farther from the largest city (than the urban area) and the community was a predominantly farming one | SBP≥140 and/or DBP≥90mmHg  Or anti-hypertensive medication use*  Or self-reported HTN diagnosis* | *P*=0.359  (x^2^ test) | NA | Multivariate logistic regression shows that  Female sex (OR=1.551; P= 0.046)  Obesity (OR = 2.8; 95% P = 0.001) were associated with increasing risk of HTN while  Increasing income was associated with decreased risk of HTN;  0.798 per unit increase in naira (OR = 0.798; P = 0.007). | Use of smokeless tobacco and alcohol more common in rural areas (P<0.007 and P=0.023 respectively) |
| Agyemang, 2006, Ghana | Residents of Ashanti region of Ghana | 1431 [NR]  (Reported as “high”) | 40.4% | 55.0%  Rural  59.0%  Urban  52.3% | 35.9 | 29.4% | [NR]  Males  27%  Females  27% | [NR]  Males 33.4%  Females 28.9% | Rural areas were villages with no “main water supply” and major occupation was subsistence farming. Urban area was the capital city in the region. | SBP $\geq$140 mmHg and/or DBP$\geq$90 mmHg,  Or anti-hypertensive medication use | Multivariable logistic regression.  Urban^ OR 2.0 (95% CI=1.5 – 2.6)  Urban Males~ OR= 1.7 (95% CI=1.1 – 2.7)  Females~ OR= 1.5 (95% CI=1.0 – 2.4) | Age, level of education, smoking, alcohol, BMI, heart rate | HTN prevalence increased with age.  Overweight and obesity associated with higher prevalence of HTN | Rural men had significantly higher rates of smoking and alcohol than urban men. Urban participants more likely to be overweight and obese |
| Agyemang,  2017,  Ghana | Adults aged 25-70 years living in rural and urban Ghana and in 3 European cities (London, Berlin, Amsterdam) | 2492 in Ghana (out of 5659 total including Ghanaians in diaspora)  {76% in rural }  {74% in urban}  None responders not described | 41.9%  (1043)  Malen=405  Female n: 638 | 67.1%  N=(1672)  (rural: 61.2% (n=638);  urban 71.3% (n=1034) | [NR]  Rural men: 46.2 (95% CI: 45.0- 47.5)  Rural women: 46.7 (95% CI: 45.7- 47.6)  Urban men: 46.5 (95% CI: 45.4-47.7)  Urban women: 44.7 (95% CI: 44.1- 45.4) | [NR] | [NR]  Men:  Age standardised: 22.7%  Crude: 22.2% (90/405)  Women: both age standardised and crude: 27.9% (178/638) | [NR]  Men:  Age standardised: 33.6% Crude:34.2% (142/415)  Women: Age standardised: 31.4%. Crude:29.4% (304/1033) | Not explicitly stated. | SBP≥140mmHg  OR DBP≥90mmHg  OR  Being on antihypertensive medication  semiautomated device (the Microlife WatchBP home Widnau, Switzerland) | Prevalence ratio of HTN in urban men: 1.37 (95% CI: 1.10–1.70)  Prevalence ratio of HTN in urban women: 0.99 (95% CI: 0.85-1.16)  **No analysis of difference between the sexes reported** | Age, BMI, level of education | none | **Prevalence reported, no statistical analysis.**  Current smoking:  Rural men: 5.8% (95% CI: 3.8–8.6)  Rural women: 0.0% (95% CI: 0.0–0.1)  Urban men: 3.3% (95% CI: 1.9–5.6)  Urban women: 0.1% (95% CI: 0.0–1.0)  **Depressive symptoms**  Rural men: 5.1% (95% CI 3.2, 7.8)  Rural women: 7.8% (95% CI 6.0, 10.4)  Urban men: 2.8% (95% CI 1.6, 5.0)  Urban women: 4.2% (95% CI 3.1, 5.6)  **Low physical activity**  **Rural men:** 10.9% (95% CI 8.1, 14.4)  Rural women: 22.3% (95% CI 20.0, 26.8)  Urban men: 22.7% (95% CI 18.8, 27.2)  Urban women: 40.6% (95% CI 37, 43.7) |
| Banigbe, 2020, Nigeria | Male partners (aged≥18 years) of pregnant women participating in a Healthy Beginning Initiative between 2016 – 2018 in Benue State, North central Nigeria | 6,538  {NR}  However, original sample was 6766, 228 persons (3.4%) excluded due to implausible BP records or age <18years.  Excluded persons not described. | 77.8%  (5,087)  Semi-urban: 18.4%  (1,204) | 0% | [NR]  Median age 31 years  (IQR: 26 – 37 years) | 23.4%  (1,527/6,538)  Self-reported history of HTN: 4.5% (297/6,538) | 23.2%  (1,178/5,087)  Semi-urban: 21.8%  (263/1,204) | 34.8%  (86/247) | [NR]  Not stated | JNC-7  SBP≥140 and/or DBP≥90  OR  Self-reported HTN and BP>140/90  [Digital Sphygmomanometer by Omrom Healthcare Inc. USA] | Multivariate logistic regression.  Urban aOR= 1.6, (95%CI: 1.2–2.1) p value= 0.001  Semi-urban aOR= 0.9 (95%: 0.8-1.1) p value= 0.2 | [NR]  Even in supplementary data | being overweight [aOR: 1.5, 95%CI: 1.3–1.8], p value= 0.001  being obese [aOR: 2.6, 95%CI: 2.0–3.3], p value= 0.001  alcohol use in the 30 days prior [aOR: 1.2, 95%CI: 1.1–1.4], p value= 0.001  Age 41-50 years [aOR: 1.4, 95% CI: 1.2–1.7], p value= 0.0004  Age >50 years [aOR: 2.4, 95% CI: 1.8–3.0], p value= <0.01 | [NR] |
| Cappuccio *et al.*, 2004, Ghana | Residents of Ashanti region of Ghana aged 40 – 75 years | 1013 {53.4%} | 47.5% | 62.0% | 54.7 ± 11.3  Rural  54.5 ± 11.2  Semi-urban 54.9 ± 11.4 | 28.7% | 24.1% | 32.9% | [NR] | SBP≥140 and/or DBP≥90 mm Hg  Or anti-hypertensive medication use | *p=*0.002  (X^2^ test) | NA | HTN prevalence was comparable in men and women (29.9% versus 28.0%)  Prevalence of HTN increased with age in both location types, and in both sexes | [NR] |
| Ejim *et al.*, 2013, Nigeria | Residents of Enugu state, south eastern Nigeria aged 40 – 70 years | 543 [NR]  (70.4% in the rural area, 40% in the urban area) | 56.7% | 68.7% | 56.3 ± 9.9 | 47.7%  Systolic HTN 37.9%  Diastolic HTN 33.7% | 45.1%  Systolic HTN 40.3%  Diastolic HTN 27.9% | 51.1%  Systolic HTN 34.9%  Diastolic HTN  41.3% | Occupation in the rural community was mostly subsistence farming. Urban community had a much larger population. | SBP≥140mmHg and/or DBP≥90mmHg  Or anti-hypertensive medication use | *p=*0.193  Systolic HTN *p=*0.212  Diastolic HTN *p=*0.001  (X^2^ test) | NA | [NR] | Abdominal and general obesity were significantly more common in urban than rural areas (P<0.0001 for both). |
| Houehanou *et al.*, 2015, Benin | Randomly selected national sample of persons in Benin aged above 24 years and below 65 years. | 6762 {99%} | 66.4% | 49.5% | 42.8 ± 0.3 | 28.4% | 27.5 % | 29.9% | Defined according to Benin’s Statis- tical and Economic Analysis Institute (INSAE) list. (2013) | SBP≥140 mmHg and/or DBP ≥90 mmHg  Or anti-hypertensive medication use | Logistic regression  Urban OR= 1.4 (95% CI 1.2 – 1.6) | Age group, gender | [NR] | Obesity, DM, were significantly higher in urban than rural areas.  Daily tobacco smoking higher in rural than urban areas. |
| Kodaman *et al.*, 2016, Ghana | Residents of Brong Ahafo region of Ghana aged ≥ 18 years | 3317 [NR]  (“few potential participants refused to engage”) | 32% | 56.6% | [NR]  (range 18 – 99 years)  Rural  Male 44.9 ± 17.2  Female 43.9 ± 15.9  Urban  Males 42.9 ± 12.6  Females 42.1 ± 11.3 | [NR] | [NR]  Males 20%  Females 21% | [NR]  Males 34%  Females 32% | Urban area was capital city of the region with population size 250,000. Rural areas were surrounding villages within 10km radius of capital city each with population size <5000 | SBP≥140mmHg or DBP≥90mmHg,  Or anti-hypertensive medication use prescribed by a physician | Multivariable logistic regression  Urban OR= 3.2 (95% CI 2.6 – 4.0) | Age, sex | Prevalence of HTN did not differ significantly with sex  Prevalence of HTN increased with age (though the marked age-related increase began a decade earlier in urban than rural cohort) | Prevalence of obesity, DM, dyslipidaemia significantly higher in urban than rural areas |
| Minicuci *et al.*, 2014, Ghana | Nationally representative sample of ageing population (aged 50 years and above) across the 10 regions of the country | 4724 {95.9%} | 59.4% | 50.3% | [NR]  (55.1% aged 50 – 64 years) | Self-reported HTN14.2%  Measured HTN 51.1% | Self-reported 8.0%  Measured HTN 45.6% | Self-reported HTN 23.1%  Measured HTN 59.2% | [NR] | SBP ≥140 and/or DBP ≥ 90  Or anti-hypertensive medication  Or  Self-reported HTN diagnosis* | Multivariable logistic regression  Measured HTN Rural OR= 0.77 (95% CI 0.61 – 0.97)  Self-reported HTN. Rural OR= 0.53 (95% CI 0.39 - 0.72) | Smoking, alcohol | Self-reported HTN was significantly higher in women (17.4% vs 11.3%, P<0.0001)  No sex difference in prevalence of measured HTN.  HTN prevalence increased with income quintile and age (though highest in 65-74 yrs group not the >75yrs group) | Heavy drinking and smoking significantly more common in rural areas.  Prevalence of obesity, DM and dyslipidaemia significantly higher in urban areas |
| Ntandou *et al.*, 2009, Benin | Persons aged 25 – 60 years who had been living in the study area for at least 6 months | 541 [NR] | 31.4% | 49.9% | [NR]  (Men  37.3 years ± 10.1  Women  39.0 years ± 10.0) | [NR] | 24.1% | semi-urban  21.6%  urban 26.5% | Defined according to Benin’s Statis- tical and Economic Analysis Institute (INSAE) list (2003). | SBP ≥ 130mmHg, DBP ≥85mmHg | Multivariable logistic regression  Semi-urban 0.36 (0.17 – 0.74)  Urban 0.38 (0.20 – 0.74) | Age | Logistic regression showed no significant sex difference in the prevalence of HTN.  High SES was associated with a 54% reduction of the likelihood of HTN (OR, 0.46; *P* < 0.05) compared to low SES | Abdominal obesity and binge drinking significantly more prevalent in urban than rural areas |
| Obirikorang *et al.*, 2015, Ghana | Residents of Ashanti region of Ghana | 672 [NR] | 53.6% | 53.6%  Rural  60.0%  Urban  46.2% | [NR]  [Median age (IQR) 50 years (39 – 58)] | 34.8% | 36.7% | 32.7% | Rural and urban areas classified based on population size, settlement, location and way of life of the people | SBP≥140mmHg and/or DBP≥90mmHg | *p=*0.2921  (Fisher’s exact test) | NA | Prevalence of HTN significantly higher in males in both rural and urban areas  Many other CVD risk factors e.g obesity, high waist circumference, physical inactivity and low HDL cholesterol were more prevalent in females | [NR] |
| Odili, 2020, Nigeria | Adults (aged 18 years and above) from 12 rural and urban communities in Nigeria from a state each in the 6 geopolitical zone.  Anambra (South-East), Akwa-Ibom (SouthSouth), Oyo (South-West), FCT-Abuja (North-Central), Zamfara (North-West), and Gombe (North-East) | 2503  Initial RR=95% (3077 of 3239) invited.  However, after exclusion for missing data and other reasons 2503 analysed [77.3%]  Responders analysed said to have similar sociodemographics with those excluded | 53.2% (1332) | 56.9%  (1425) | 43.8 ± 16.2 years | 32.0%  (802) | Overall calculated from table: 31.5% (420/1332)  South-East: 60% (15/25)  South-South:46.5% (112/241)  South-West: 41.3% (52/126)  North-Central:18.0%(9/50)  North-West: 27.2% (73/268)  North-East: 25.6% (159/622) | Overall calculated from table: 32.6% (382/1171)  South-East: 53.6% (75/140)  South-South: 37.7% (40/106)  South-West: 39.4% (115/292)  North-Central: 20.7% (25/121)  North-West: 15.6% (42/270)  North-East: 35.1% (85/242) | Each state is made up of local government areas, and **administrative headquarters of each is regarded as an urban area** according to the **National Urban Development Policy**  **Rural not defined, but said to have mostly subsistence farmers.** | Office systolic BP greater ≥ 140 mm Hg or diastolic BP greater ≥ 90 mm H or reported use of antihypertensive medication.  Accoson® mercury sphygmomanometer.  An individual's blood pressure was the average of 5 readings. | None  X^2^ reported to have been used for categorical variables, but no analysis of the rural vs urban sites | Not applicable | Not reported | Higher 24 hrs urinary excretion of sodium in urban compared to rural (114.9 vs 86.0 mmol) p<0.05 |
| Odland,  2020, Sierra Leone | Individual over 40 years randomly selected in a household survey based on WHO STEPS survey | 2071  {NR} | 62.9%  Calculated N= 1302 | 49.0%  Calculated N= 1015 | [NR]  Median 51.0 years (IQR 45.0 to 63.0 years) | 49.6%  Calculated N= 1027 | 46.0%  N= 599/1302 | 55.8%  Calculated N= 429/769 | From Sierra Leone 2015 national census data. Urban areas defined as “as a locality of 2,000 or more people” from the census website and documents (referenced in the paper) | SBP≥140mmHg  OR  DBP≥90mmHg  OR  History/self-report of taking antihypertensive medication in the last 2 weeks.  Omron M6 AC LED blood pressure monitor | Multivariable logistic regression  OR for HTN in urban area 1.04 (95% CI 1.01 – 1.08)  P=0.014 | Sensitivity analysis done with Obesity (BMI >30) | Sex, Male OR for HTN = 0.78 (95% CI 0.75 to 0.80) P<0.001  Increasing age  Any education OR= 1.17 (95% CI =1.14 – 1.21), p<0.001  Marital status: Married or cohabiting (compared to being single/divorced/widowed) OR= 0.8 (95% CI= 0.78- 0.83), p <0.001  Wealth quintile: mixed effect, initial decreased risk as wealth quintile increases, then an increased risk in later quintiles | **Higher in urban:**  DM OR= 1.46 (95% CI= 1.34-1.60), p<0.001  Overweight/Obesity OR= 1.17 (95% CI= 1.12-1.21), p<0.001  Smoking OR= 1.13 (95% CI= 1.08-1.17), p<0.001  **Lower in urban:** Dyslipidaemia OR= 0.84 (95% CI= 0.75-0.93) |
| Ogah *et al.*, 2013, Nigeria | Residents of Abia state, Nigeria aged ≥ 18 years | 2928 {99.5%} | 47.0% | 52.2% | 41.7 6 ± 18.5 | [NR]  Systolic HTN 31.4%  Diastolic HTN 22.5% | [NR]  Systolic HTN  Men 33.5%  Women 30.5%  Diastolic HTN  Men 23.4%  Women 25.4% | [NR]  Systolic HTN Men 33.6%  Women 26.4%  Diastolic HTN Men 20.6%  Women 18.4% | Rural and urban communities defined based on the standard criteria adopted by the Nigerian National Population Council | SBP≥140mmHg and/ or DBP ≥ 90mmHg  Systolic HTN SBP≥140mmHg and DBP<90mmHg  Diastolic HTN SBP<140mmHg and DBP≥90mmHg | [NR] | NA | Predictors of HTN (using logistic regression models) were age, obesity and pulse rate. | [NR] |
| Oguoma *et al.*, 2015, Nigeria | Residents of Delta and Lagos states aged ≥ 18 years | 422 [NR] | [NR]  (percentages from the two rural locations  Abbi 34.4%  Kwale 42.9%) | 64.7% | [NR]  (Females 42.9 ± 20.7  Males 38.3 ± 20.5) | 35.7% | [NR]  Abbi 37.3%  Kwale 23.3% | 53.3% | [NR] | SBP ≥ 130mmHg and/or DBP ≥ 85mmHg | [NR] | NA | Prevalence of HTN progressively increased with increasing age groups  Prevalence of HTN significantly higher in females than males | [NR] |
| Okello**,  2020, Nigeria | Adults aged 18 years and above  SevenCEWA study  Included 3 rural and 1 “semi-urban” sites in Nigeria | 2065 from Nigeria  Total cohort 3549  {100% resoonse rate from the Nigerian sites, although 91% for the whole study cohort) | 76.3%  (1576) | 56.3%  (1163) | Not applicable  Ogane-Uge: 39.2years ±19.5  Okpok Ikpa: 38.5years ±14.0  Olorunda Abaa: 41.2years ±12.9  Semi-urban: Ikire: 48.1years ±18.1 | N/A as overall prevalence included data from the East African sites (Tanzania and Kenya) | Ogane-Uge (**total n=403**), 33.0% (95% CI: 28.4, 37.7)  Age-standardised 27.7% (95% CI: 24.4, 30.9)  Okpok Ikpak (**total n=465**) 20.4% (95% CI: 17.9, 25.6)  Age standardised 12.6% (95% CI: 11.2, 14.1)  Olorunda Abaa (**total n=708**) 23.3% (95% CI:20.3, 26.6)  Age standardised 20.8% (95% CI:18.4, 23.2) | Semi-urban  Ikire (**total n= 489**)  38.6 % (95% CI: 34.2, 43.0)  Age standardised 27.5 (24.6, 30.4) | Not explicitly stated | JNC 7  SBP ≥140 mmHg  and/or  DBP ≥90 mmHg  and/or  self-report of previous diagnosis with or without current treatment with antihypertensive medications  OMRON-Healthcare-Co HEM-7211-E-Model-M6; Kyoto, Japan | Formal statistical analysis comparing rural and semi-urban sites not done?  **Gender based analysis also done but for full data set including East African sites and Nigerian data cannot be extracted from the analysis** | Not applicable | Some reported but analysis from Nigerian sites cannot be extracted out of the others i.e Kenya, Tanzania | Overweight/Obesity and self-reported DM higher in semi-urban compared to rural sites but no formal analysis of the difference done.  Mixed pattern for smoking and alcohol use. |
| Okpechi *et al.*, 2013, Nigeria | Residents of Abia state, south eastern Nigeria aged ≥ 18 years | 2983 {99.5%} | 53.2% | 52.1% | 41.7 (SEM = ±0.3) | 31.4% | 32.0% | 30.7% | [NR] | SBP≥140 mmHg and or DBP≥90 mmHg  Or anti-hypertensive medication use | *p>*0.05  (Pearson correlation coefficient) | NA | Prevalence of HTN significantly higher in males than females in both rural and urban areas (34.9% vs 28.1%; P<0.05) | Obesity and overweight significantly higher in urban compared to rural areas. |
| Oyekale, 2019, Ghana | Women aged 15-49 years in a nationwide Demographic and Health Survey (DHS) in 2014 | 9367  [99.7% if using the number that participated in the survey 9396]  [97% if using the total number of eligible participants 9656]  Nonrespondents not described | 46.2% (4329) | 100% | {NR}  Age range 15-49 years  Majority of participants were less than 40 years. Majority of HTNsives were above 30 years | 13.28%  (1244) | 9.73% (421) | 16.34% (823) | According to the Ghana DHS,  **However, review of the DHS did not reveal explicit definitions for rural and urban** | medically confirmed to have raised blood pressures that require regular intake of some medications and those for which the average values of their last two blood pressure measurements for systolic or diastolic were ≥140 mm Hg or ≥90 mm Hg, respectively.  **BP measurement device not stated** | None  Probit regression model of obesity on hypertension.  Risk lower in rural | NA | Overweight/obesity associated with increasing risk of HTN  Other factors that influencing HTN were age of women, region of residence, urban/rural residence (lower risk in rural), being pregnant (lower risk in those pregnant), access to medical insurance, currently working, consumption of broth cubes, processed can meats, salted meat and fruits. | {NR} |
| Seck *et al.*, 2014, Senegal | Residents of St Louis, Senegal who are aged ≥18 years and above living in the study area for at least 3 months | 1037 {99%} | 47% | 60% | 48.0 ± 16.9  Rural  43.5 ± 17.2  Urban  51.6 ± 15.7 | 39.1% | 33.8% | 43.3% | [NR] | SBP ≥140mmHg and/or DBP ≥90mmHg  or  any prescription of anti-hypertensive medication in the past 2 weeks,  or any self-reported history of hypertension. | *p=*0.002  (Pearson X^2^ test) | NA | [NR] | Obesity (general and central) and diabetes are significantly higher in urban compared to rural population  Alcohol use and physical inactivity significantly higher in rural compared to urban areas |
| Soubeiga *et al.*, 2017, Burkina Faso | Nationally representative sample of persons aged 25 to 64 years who had been residing in the country for at least six months on the day of the survey | 4629 {96.4%} | 72.2% | 54.2% | [NR]  (41.98% aged 25 – 34 years) | 18% | 15.37% | 24.81% | [NR] | SBP≥140mmHg  and/or DBP ≥90mmHg  Or anti-hypertensive medication use | *p<* 0.001  (Fisher’s exact test) | NA | Prevalence of HTN increased with age and BMI in both rural and urban areas.  Additionally, in rural areas male sex, high fat intake, family history of HBP and low level of HDL cholesterol were associated with increasing odds of HTN | [NR] |
| Umuerri, 2020, Nigeria | Adults aged ≥18 years from two communities in Delta State who have lived in the study sites for at least one (1) year. | 852  [NR]  (total sample was 866, 14 persons excluded from this analysis due to implausible BP readings. Their basic xtics compared to included participants not stated | 44.2%  N=377 | 55.9%  N=476 | 42.64 (±16.07) years | 29.3%  N= 250/852 | 21.8%  Calculated N= 82/377 | 35.4%  Calculated N= 168/475 | Rural (Jesse) and urban (Warri) sites were 47km apart. Whereas Jesse is sparsely populated and majorly agrarian, Warri is densely populated and the commercial hub of Delta State, especially as it relates to the oil and gas industry. | JNC 7  SBP≥140mmHg  OR  DBP≥90mmHg  OR  self-reported a history of HTN as diagnosed by a healthcare professional irrespective of their current BP readings or use of anti-HTNsive drugs  Omron BP-785 Intellisense automated sphygmomanometer | Chi-square  (χ2 = 30.480, df=2, p<0.001)  Spearman’s Rank correlation coefficient= -0.188 p <0.001 | N/A | BP category significantly correlated with age (more risk with increasing age, CC =0.243 p<0.001*), level of education , employment status, body mass index (obesity and overweight increased the risk χ2 = 70.210, df = 6, p <0.001) and fruit intake less than five portions daily  ****no correlation with sex, although a females accounted for a higher proportion of HTNsives (and pre-HTNsives)** | Overweight/obesity more prevalent in urban {(overweight n=112 (23.6%); obesity n= 73 (15.4%)} compared to rural {(overweight n= 63 (16.7%), obesity n= 20 (5.3%)  p<0.001 |
| Van der Sande *et al.*, 2000, Gambia | Persons aged ≥ 15 years who had been residing in the country for at least six months on the day of the survey | 5389 {78.1%} | 59.8% | 58.1% | 35.4 | BP ≥160/95 mmHg 7.1%  BP≥140/90mmHg 18.4% | BP≥160/95 6.8% (plus those on medication 6.9%)  BP≥140/90 17.7% (plus those on medication17.8%) | BP≥160/95 7.5% (plus those on medication 8.9%)  BP≥140/90 19.4% (plus those on medication 20.3%) | Main occupation in the rural area was subsistence farming, while in the urban areas (including the country capital) it included white collar jobs, and artisans, trade and industry work | SBP≥160mmHg and/or DBP ≥95mmHg  Borderline HTN SBP≥140mmHg and/or DBP ≥90mmHg | Multivariable logistic regression  Urban OR=1.8 (95% CI 0.9 – 3.5) for BP ≥ 160/95mmHg  Urban OR= 2.0 (95% CI 1.2 – 3.2) for BP ≥ 140/90mmHg | Age, sex | Prevalence of HTN significantly higher in males compared to females in both rural and urban areas using a BP of ≥ 140/90mmHg but not using a BP of ≥160/95mmHg | Obesity, diabetes, hyperlipidaemia and physical inactivity were significantly less prevalent in the rural population than the urban.  smoking was more common in the rural than in the urban study population |

** rural and semi-urban participants only. No urban participants. ^ includes data from outside west Africa.

[NR]= Not reported; N/A= not applicable; BP= blood pressure; SBP= systolic blood pressure; DBP= diastolic blood pressure; HTN= hypertension; SEM= Standard error of mean; SD= Standard deviation;
