# Supplementary material for: Rural-urban difference in the prevalence of hypertension in West Africa: a systematic review and meta-analysis
Source: J Hum Hypertens. 2022 Apr 16;38(4):352–64. doi: 10.1038/s41371-022-00688-8 (PMC11001577; doi:10.1038/s41371-022-00688-8)
Supplement: Supplementary file 8 — Supplementary Material 8 [file 41371_2022_688_MOESM8_ESM.pdf]

S8 - Caveats for meta-analysis

The odds ratio is calculated by noting the number of positive and negative cases.

In this instance prevalence is provided.

Therefore, the number of cases is subtracted from the denominator (rneg=rdenom-rpos)

columns

rpos: number of positive rural hypertension cases

rdenom: rural denominator

rneg: number of rural non cases (i.e. rdenom-rpos)

upos: number of positive urban hypertension cases

udenom: urban denominator

uneg: number urban rural non cases (i.e. udenom-upos)

notes:

#Agyemang, 2006, Ghana

No prevalence noted but males and female data provided so these were added together (rural = 64/237 + (92/341) = 156 / 578 , urban (136/407) + (129/446) = 265 / 853

#Agyemang, 2017, Ghana

No prevalence noted but males and female data provided so these were added together (rural = 90/405 + 178/638 = 268/ 1,043, urban (142/415) + (304/1033) = 446/1448

#Banigbe, 2020, Nigeria

This includes semi-urban. For the purposes of this analysis, semi-urban and urban are grouped together < - need to decided is this is appropriate

Urban (86/247) + semi urban: (263/1204) = 349/1451

#Cappuccio, 2004, Ghana

semi-urban counted as urban

#Kodaman, 2016, Ghana

No prevlaence noted but males and female data provided so these were added together:

Rural: Males 94/469+ females 122/583 = 216 / 1052

urban: Males: (330/972) + Females: (414/1293) = 744 / 2265

#Minicuci, 2014, Ghana

Two prevalences provided. self-reported vs measured.

Rural: Self-reported HTN 8.0% (224/2806) Measured HTN 45.6%

(1280/2806)

Urban: Self-reported HTN 23.1% (443/1918) Measured HTN 59.2%

(1135/1918)

measured used in meta-analysis

Rural: 1280/2806, urban 1135/1918

#Ntandou, 2009, Benin

This includes semi-urban . For the purposes of this analysis, semi-urban and urban are grouped together < - need to decided is this is appropriate

semi-urban (37/171) + urban (53/200) = 90 / 371

#Ogah, 2013, Nigeria

Systolic and diastolic hypertension measured separately in both males and females

For purposes of this meta-analysis systolic HTN in combined males and females used:

Rural: Systolic HTN Men (238/710) + Women (257/843) = 495 / 1553

Urban: Systolic HTN Men (232/689) + Women (181/686) = 413 / 1375

#Oguoma, 2015, Nigeria

Multiple regions provided for rural. These are added together.

Rural: Abbi (54/145) + Kwale (42/181) = 96 /326

#Okello, 2020, Nigeria

Multiple regions provided for rural. These are added together.

Ogane-Uge: (133/403) + Okpok Ikpak (95/465) + Olorunda Abaa (165/708) = 393 / 1576

Semi-urban counted as urban for this analysis: 189 / 489
